# Theory of mind goes to school: Does educational environment influence the development of theory of mind in middle childhood?

**DOI:** 10.1371/journal.pone.0237524

**Published:** 2020-08-14

**Authors:** Joanna Smogorzewska, Grzegorz Szumski, Paweł Grygiel

**Affiliations:** 1 University of Warsaw, Faculty of Education, Warsaw, Poland; 2 Jagiellonian University, Faculty of Philosophy, Cracow, Poland; Jiangsu Normal University, CHINA

## Abstract

Previous research has shown that the development of theory of mind (ToM) depends on various individual and social factors, but very little research has examined the role of the natural educational environment in the development of ToM in middle childhood.

In accordance with the importance of social factors in development, in this longitudinal study of 156 typically developing children, we investigated whether educational setting—classes containing children with disabilities (inclusive) or without such children (general education)—is associated with enhanced ToM development. ToM was measured with the ToM Scale, the Chocolate task and the Faux Pas Recognition Test. Analysis showed that ToM development was better among children educated in inclusive classes than among those educated in traditional classes. The results have implications for ToM development among children with and without disabilities as well as for educational practice.

## Introduction

Theory of mind (ToM) is a very popular concept that can be defined as the understanding that all people possess mental states—such as desires, emotions, beliefs and intentions—that cannot be directly observed but that can be used to make predictions about, e.g., how other people will behave [1, 2]. ToM is a developmental ability, meaning that children—from early years, through middle childhood and adolescence to adulthood—change and develop their understanding of self and others, as well as ambiguous situations and emotions and sarcasm. With age this understanding becomes more developed and encompasses not only well-known, but also new and unexpected situations [1, 2].

Recent publications have indicated that ToM has “grown up”[3]; in other words, investigation of ToM in children in middle childhood is a manifestation of a current knowledge that ToM abilities continue to develop into adulthood (e.g., [4, 5]). Given this shift, there is a need to extend the contexts in which ToM is investigated to include milieus such as schools in addition to the family environment. This idea is in line with theories, such as the bioecological model of child development [6] showing the important role of social factors and interactions in children’s development (see also: [7]). Analyses show that not only family but also school, peers and reciprocal relationships are meaningful for children’s development (e.g., [8]). Different characteristics of a classroom, such as its quality and organization, influence each other and impact children’s development [9]. Research has shown that the quality of the classroom environment is important for different areas of children’s functioning, such as socioemotional development and academic achievement [9, 10].

Therefore, the central topic of this paper is the relationship between the school environment and ToM development. We investigated whether ToM develops better in an inclusive educational environment (i.e., one in which children with disabilities are educated alongside children without disabilities) than in the general educational environment (i.e., one that excludes children with disabilities), i.e., whether being educated in an inclusive classroom is associated with enhanced theory of mind development.

### Theory of mind and school

To date, there have been few studies on the relationship between school environment and ToM development. Some of the existing studies [11–13] focused on ToM training that was carried out in school but not as part of regular lessons. The results showed that in middle childhood, participating in social interactions that require an understanding of emotions promotes ToM development [13]. It was also shown that directing children’s activities toward understanding the mental states of other people and the development of language for describing the emotions, thoughts and intentions of others fostered ToM development in middle childhood [11, 12, 14].

Thus far, only one study has tested the hypothesis that ToM development can be influenced by the nature of the educational environment [15]. Overall, it was shown that in schools based on the constructivist approach, children’s ToM developed faster in comparison to that of children in the traditional Chinese school.

### Inclusive education and theory of mind development

Inclusive education involves providing students with disabilities access to mainstream schools and academic programs [16]. There is evidence that the achievement and social development of children without disabilities are better when they are educated in inclusive classes rather than in traditional classes [17, 18]. The results showing that inclusive education is beneficial for children’s social and cognitive development can form the basis for the hypothesis that inclusion is important for ToM in terms of sociocognitive ability. To date, however, we are not familiar with research that has examined such a hypothesis.

An important feature of inclusive schools and classes is that teachers make an effort to create a community that includes *all* students and to inculcate positive attitudes toward diversity using various strategies and group activities to prevent the social exclusion of students with disabilities (e.g., [19]). A valuable strategy is to promote a group identity and group norms that involve social acceptance of peers with disabilities and prosocial behavior toward them [20]. One way of accomplishing this is to employ affective interventions designed to have a positive influence on attitudes toward peers with disabilities and promote prosocial attitudes [21]. Such interventions are delivered to entire cohorts of children and can include activities such as reading positive, realistic stories about people with disabilities, showing examples that teach children about different kinds of disabilities, and discussing the specific needs of people with disabilities and ways of fulfilling them. Research shows that such activities improve children’s attitudes toward peers with disabilities [22]. The results of experimental studies indicate that activities similar to those mentioned above develop ToM among children (e.g., [23]). Teachers in inclusive classrooms are also encouraged to use peer-mediated (PM) strategies to improve relations between students with and without disabilities. This approach involves training some students in each class to better understand peers with disabilities and acquire the skills needed to have effective and satisfying interactions with them (e.g., [24]). This kind of training is designed to improve the participants’ understanding of the needs and emotions of others, so it can be thought of as a type of ToM training. The effects of training some students should generalize to other students, who then imitate the behavior of their trained peers [25].

In an inclusive environment, there are many natural opportunities for students without disabilities to interact with peers with disabilities. Some students voluntarily engage in such relationships and frequently interact with peers with disabilities, which have benefits for their social development, i.e., improvement of responsiveness to others’ needs and development of the ability to understand others [26]. According to the theory of Carpendale and Lewis [27], such social behaviors can influence ToM development.

Peer interactions are also an opportunity to display prosocial behaviors toward others. There is evidence, reported in a meta-analysis, of a positive, bidirectional relationship between ToM and prosocial behaviors [28]. Constant contact and interactions with children with special needs and disabilities can foster prosocial behaviors. Research has shown that people with a particular need for help and care are the most common target of prosocial behavior [29]. Thus, an inclusive educational environment presents children with many opportunities to display a positive attitude toward others. A naturally diverse classroom provides a good environment for learning about differences between people and hence for ToM development. Nevertheless, teachers can increase the opportunities for interactions between students through lesson arrangements and the use of group activities such as cooperative learning and peer tutoring, because these activities improve academic achievement and social relations between students (e.g., [30]).

### The current study

The aim of this study was to investigate whether educational homogeneity in the educational environment (inclusive education, i.e., heterogeneous, vs. general education, i.e., homogeneous) is associated with enhanced ToM development in middle childhood in children without disabilities. We assumed that ToM development would be promoted by (1) everyday interactions between children with and without disabilities, (2) the development of prosocial behaviors, and (3) the teaching of social norms such as tolerance and acceptance of peers, as these would help children understand the emotions, needs, desires and beliefs of others. We therefore hypothesized that learning in an inclusive educational environment enhance students’ ToM development in comparison to a traditional educational environment from which children with disabilities are excluded.

## Methods

### Participants

The study was approved by the ethics committee of the Maria Grzegorzewska University (approval number 60-2014/2015). Parents provided written consent for their children’s participation, and the children agreed verbally to take part in the study.

The sample for this longitudinal study consisted of 156 typically developing children attending primary schools in Poland. At the time of the first assessment the children were in first or second grade (children in Poland can start school at 6 or 7 years old depending on their cognitive, social and emotional development and their parents’ decision). In Poland, there are two major settings in which typically developing children may be educated: (a) general education, in which classes do not include any children with disabilities and (b) inclusive education, in which classes include no more than 5 children with different kinds of disabilities, and the children are taught by two teachers—a mainstream teacher and a special education teacher. The merit of having a special education teacher permanently available in the classroom is a topic of debate. Although the special education teacher can provide valuable support to children who need it and initiate and implement programs designed to foster social interactions between children with and without disabilities (e.g., [31]), his or her presence may inadvertently contribute to the stigmatization of children with disabilities because it suggests that they cannot manage alone [32], and a lack of interaction between different groups of children can negatively influence ToM development.

The sample included children from all regions of Poland attending a random selection of schools and classes (drawn from the Polish Educational Information System base). Children were recruited according to the following scheme: (a) we contacted the sampled school and its principal—the principal needed to agree for the study to be conducted at school; (b) after obtaining the principal’s agreement, we contacted teachers from all relevant classes at the school; (c) after the teachers agreed they received all necessary information; (d) parents received information about the study along with a request for their child’s participation. After taking part in all three waves of the study, children received gifts—picture books—as an acknowledgement for participation in the study. During the course of the study, 6 families dropped out, in most cases because the child moved to another city or school.

Detailed information about the sample is presented in Table 1.

**Table 1:** Detailed characteristics of participants.

	Whole sample	Inclusive classroom	General education classroom
Sample	156	66 (42.31%)	90 (57.69%)
Sex			
Boys:	87 (55.8%)	40 (60.6%)	47 (52.2%)
Girls:	69 (44.2%)	26 (39.4%)	43 (47.8%)
Age	*M* = 7.5, *SD* = 0.65, range: 6.0–9.0	*M* = 7.59, *SD* = 0.63, range: 6.01–8.09	*M* = 7.52, *SD* = 0.66, range: 6.00–9.00
City of residence:			
> 100 000 inhabitants:	89 (60.2%)	43 (68.3%)	46 (54.1%)
20 000–100 000 inhabitants:	34 (23.0%)	13 (20.6%)	21 (24.7%)
Up to 20 000 inhabitants:	25 (16.9%)	7 (11.1%)	18 (21.2%)
Number of parents with college or higher education	Mothers: 98 (66.3%)	Mothers: 43 (68.2%)	Mothers: 55 (64.7%)
	Fathers: 76 (55.1%)	Fathers: 32 (54.3%)	Fathers: 44 (55.7%)

Almost all the classes taking part in the study were taught by women; there was only one male teacher working in an inclusive class. There were between 19 and 25 students in most classes, and children from twenty-six inclusive classes and 37 traditional classes participated in the study.

### Measures

#### Theory of mind

ToM was assessed using the Theory of Mind Scale (ToM Scale; [33–35]), the Chocolate task [36] and the Faux Pas Recognition Test (FPRT; [37]). We used Polish versions of the ToM Scale, the Chocolate task and the FPRT prepared with the back-translation procedure.

The ToM Scale [33–35] consists of six tasks presented as short stories: diverse desires (checking whether the child understands that another person can have different desires than s/he has), diverse beliefs (checking whether the child understands that another person can have different beliefs than s/he has), knowledge access (checking whether the child understands that without looking inside a closed box, a person cannot know what is inside), false beliefs (checking whether the child understands that a person’s belief can be false), hidden emotions (whether the child understands that it is possible for a person to feel emotions internally without showing them externally to others) and sarcasm (whether the child understands that some statements should not be interpreted literally, but can be sarcastic). The Scale shows the developmental character of ToM and has been used with children of preschool and in primary school age with and without disabilities (e.g., [34, 38]).

The Chocolate task [36] measures understanding of a second-order false belief and is a simpler version of the Sullivan, Zaitchik, and Tager-Flusberg [39] task. The Chocolate task is constructed in a similar way as the tasks in the ToM Scale, which is why we decided to add it to the ToM Scale. This decision seems to be justified because children who took part in our study were older than those who are most often examined with the ToM Scale tasks, and the Scale alone could be too easy for them (the ceiling effect). The Chocolate task’s main aim is to check whether the child understands the second-order false belief, and the main asked question asked is what one character believes about what the other character believes.

The FPRT [37] has previously been solved by 7- to 11-year-olds. The original test consists of 10 stories with a faux pas and 10 stories without (control stories), but in the present study, we used 5 stories with and 5 stories without faux pas. Because all stories measure the same type of abilities, we decided to reduce the number of stories used in each wave, especially as the FPRT was not the only measure used to assess ToM. The stories for the first wave were chosen on the basis of a pilot study, in which we used all stories with children (the pilot involved s separate group of children in a similar age range to that of participants in the current study). An analysis of the results revealed which stories are difficult, semi-difficult and easy for children. For the current study, therefore, we choose the following for the first wave (similarly for stories with and without faux pas): 2 stories that were difficult, 1 that was semi-difficult, and 2 that were easy and we followed this rule during subsequent waves. The faux pas involves one of the characters in the story behaving inappropriately, which confuses the other character, but the character, who commits the faux pas does not realize that his or her behavior is inappropriate (e.g., (1) the person is presented with a pie that is made especially for him and says that he likes all kinds of pies except apple pies, but the person does not know that the prepared pie is an apple pie; (2) the person says to the other person that he does not like a specific toy, and he does not remember that the toy was a gift from the other person; (3) a person says that she does not know “this nice boy”, but the child is a girl, etc.). Four questions are asked about each story, concerning faux pas (did somebody say something inappropriate, and, if yes, what it was), memory (a specific question checking whether the child remembers what happened in the story), and understanding that the inappropriate behaviors were not purposeful) (see [37]) The FPRT is considered a more advanced ToM test in comparison to most of the ToM Scale tasks, as it concerns the second-order false belief. The reliability of the FPRT at the first assessment time was as follows: for stories with faux pas: *α* = .75, for stories without faux pas: *α* = .67) [38].

In each assessment, the children responded to questions in the ToM Scale and the Chocolate task as well as to five stories containing a faux pas and five stories without a faux pas. In the second and third assessments, we used the ToM Scale and the Chocolate task without changes and a mixture of old and new stories (test and control stories) from the FPRT. The children received one point for a story if they answered all the questions in the story correctly; otherwise, they received 0 points. In the case of each story, correct answers are linked with the story itself. Both measures have keys showing correct answers, which were used in the current study. In all the assessments, the range of possible scores was 0 to 7 in the case of the ToM Scale and the Chocolate task and 0 to 10 points in the case of the FPRT.

#### Further children and classrooms’ characteristics

Children’s perceptions of the classroom climate were assessed using the Climate of Inclusive Classrooms questionnaire [40]. This questionnaire consists of 32 statements (concerning original themes such as: Physical environment, Clear structure, Meaningful communication, Differentiation, Collaboration, Influence, Participation, and Responsibility) to which responses are given using a four-point Likert scale. The instrument was translated into Polish using the back-translation procedure. The Polish version has acceptable reliability: *α* = .83. In the current analysis, we focus on the 13 statements concerning peer interactions—for example, “In my class, we are good at listening to each other”, or “In my class we do projects together”, as we are primarily interested in contact between children. The range of possible scores was between 32 and 128 for the whole questionnaire, and the range for peer interactions statements was from 13 to 52 points.

School and peer integration and motivation to learn were assessed using a Polish version of the German questionnaire Fragebogen zur Erfassung von Dimensionen der Integration von Schülern FDI 4–6 (Students Integration Questionnaire, SIQ; [41]) in a Polish adaptation [42]. The SIQ consists of 45 statements to which responses are given using a four-point Likert scale. The SIQ is split into three subscales: social integration (satisfaction from contacts with peers), emotional integration (emotional attitude toward school), and motivational integration (motivation to learn and self-assessment of cognitive abilities). In our sample, the SIQ had acceptable reliability (overall score: *α* = .91; social integration: *α* = .84; emotional integration: *α* = .91; motivational integration: *α* = .84). The range of possible scores for each subscale was 15 to 60 points.

Academic ability was assessed using tasks from a Polish tool, the School Abilities Test (Test Umiejętności na Starcie Szkolnym, TUNSS; [43]), which is aligned with the Polish curriculum. In the first assessment, the children completed 26 tasks (14 math; 12 language), in the second, they completed 29 tasks (15 math; 14 language) and in the third, they completed 27 tasks (16 math; 11 language). In most cases these were very short tasks, completing most of them took 10–30 sec, and only a few of the tasks were longer and required 1–2 min to complete. A mix of old and new tasks were used in the second and third assessments. The ranges of possible scores for the first, second and third assessments were 0–32, 0–35 and 0–40, respectively. The test had acceptable reliability: first assessment: *α* = .92; second assessment: *α* = .93; third assessment: *α* = .95.

To conclude, during each wave children were asked to complete three tasks (two connected with theory of mind and one with school achievement) and two questionnaires. A session with one child in most cases was from 40 min to 1 hour long. The session with each child was divided into two meetings to limit the potential for the child’s fatigue (a researcher was responsible for monitoring the child’s state). The order of tasks was the same for all children to maximize the diversity of tasks following each other (the aim was to avoid boring the children).

Children’s social skills were assessed by their teachers using the Taxonomy of Problematic Social Situations (ToPSS; [44]) in a Polish translation that has been used in other studies [45, 46, 38]. The ToPSS assesses how problematic a given social situation is for a child. It consists of 44 statements to which responses are given using a five-point Likert scale. In our analyses, we reversed the scoring, so higher scores indicate better social skills. Overall, ToPSS scores range from 1 to 5 (average score). In our sample, the ToPSS had high reliability (*α* = .97).

Procedure. The children solved the tasks and completed the questionnaires individually in a quiet room in their school. An experienced educator or psychologist who had been trained to help with the data collection was present when the children were carrying out activities for the study. The children were assessed three times at 10-month intervals, i.e. the first wave was conducted in November 2015, the second wave was conducted in September 2016, and the third wave was conducted in June 2017.

Statistical analysis. The preliminary analysis, concerning possible differences between groups in the case of demographic and classrooms’ characteristics, was performed using ANOVA (comparison between groups) with repeated measures.

The main analysis was conducted with the latent trajectory model (cf. [47, 48]). The starting point of the model is an estimation of individual changes at the level of the studied variable as a function of time and thereafter an estimation of the average (mean) trajectories of these changes. The basic parameters of the model are the intercept (an initial state) and the slope (trend/pace of change). The intercept is the mean level of the analyzed variable in the first measurement time—the mean interindividual initial state. The slope is the mean change at the level of variable between the next measurement times.

The latent trajectory model can be used to analyze linear slopes (when there are at least three extended measurement times). It can, however, be easily widened, allowing the recognition of different quadratic or cubic trends (when there are a sufficient number of measurement times). The model’s limitation, however, is the need to have a considerable number of measurement times for nonlinear changes estimation (at least four for quadratic changes).

The piecewise latent trajectory model offers a solution to this problem [49]. In the piecewise latent trajectory model, nonlinearity is modeled by taking into account two (or more) slopes that reflect the trajectories before and after a chosen point (within specific pieces). In our analyses, we split the time span into two parts at the point of the second assessment time. The first part included the first and second assessment times, and the second part consisted of the changes between the second and third assessment times. Using this method, we were able to estimate not only a linear model but also a quadratic (nonlinear) model.

Statistical analyses were performed with Mplus 8.1 [50] and the Bayesian estimator [51]. We used Bayesian statistics because they perform well in small samples and are robust to nonnormality in the data [52]. In the case of the Bayesian estimator, to assess the goodness of fit of the model, we used the posterior predictive p-value (PPP; [53]) and the deviance information criterion (DIC; [54, 55]). The ideal PPP-value is .5, and values approaching or below .05 suggest poor fit. The DIC is, in turn, a useful measure for model comparison. Its interpretation is similar to that of the AIC and BIC measures—models with relatively lower levels of DIC are preferred.

For both measures (ToM Scale with the Chocolate task and the FPRT), we compared a linear growth curve (i.e., single growth process) with a piecewise growth model (i.e., two growth processes accounting for potentially nonlinear transition).

To assess group differences in the model parameters (mean levels/intercepts and change/slope), we used a multigroup design with two groups: inclusive education and general education. In these multigroup analyses, we allowed all model parameters (intercept and slope means) to be freely estimated for each group and used the Model Constraint statement to test for differences in the parameter values between groups. All missing data were estimated with full information maximum likelihood (FIML) estimation [56].

## Results

### Preliminary analysis

Table 2. presents the descriptive statistics used to compare classes with and without students with disabilities.

**Table 2:** Mean results in the ToM Scale and Chocolate task.

	Whole sample			Inclusive classroom			General education classroom		
I assessment									
*M* (*SD*)	4.35 (1.43)			4.23 (1.39)			4.44 (1.46)		
Range	0–7			0–7			1–7		
Variance	2.04			1.93			2.14		
II assessment									
*M* (*SD*)	5.11 (1.34)			5.11 (1.43)			5.11 (1.28)		
Range	1–7			1–7			1–7		
Variance	1.80			2.03			1.64		
III assessment									
*M* (*SD*)	5.91 (1.02)			6.08 (.86)			5.79 (1.11)		
Range	1–7			4–7			1–7		
Variance	1.05			0.74			1.24		
	Whole sample			Inclusive classroom			General education classroom		
Social integration with school									
	I wave	II wave	III wave	I wave	II wave	III wave	I wave	II wave	III wave
M (SD)	49.31 (8.37)	49.13 (7.68)	49.18 (8.58)	48.52 (8.11)	48.67 (8.41)	47.70 (8.93)	49.91 (8.55)	49.48 (7.11)	50.22 (8.23)
Range	24–60	23–60	18–60	24–60	23–60	18–60	25–60	25–60	22–60
Emotional integration with school									
M (SD)	46.21 (11.27)	43.83 (12.04)	43.64 (12.05)	43.04 (12.24)	42.63 (11.98)	40.38 (11.90)	48.58 (9.91)	44.75 (12.07)	45.93 (11.69)
Range	15–60	18–60	15–60	15–60	18–60	15–59	26–60	18–60	15–60
Motivational integration with school									
M (SD)	48.10 (7.91)	47.69 (7.12)	48.17 (6.31)	46.55 (7.08)	47.13 (6.60)	46.43 (6.56)	49.26 (8.32)	48.13 (7.51)	49.40 (5.86)
Range	23–60	30–60	31–60	29–60	30–60	31–60	23–60	30–60	35–60
Achievement in language									
M (SD)	15.32 (1.70)	16.77 (1.22)	17.49 (2.43)	15.23 (2.04)	16.82 (1.19)	17.10 (2.28)	15.41 (1.32)	16.74 (1.25)	16.99 (1.62)
Range	4–17	12–19	8–21	4–17	14–19	6–19	10–17	12–19	12–19
Achievement in mathematics									
M (SD)	13.33 (1.86)	14.88 (1.33)	17.49 (2.43)	12.85 (2.19)	14.64 (1.62)	17.11 (2.54)	13.69 (1.50)	15.07 (1.04)	17.75 (2.33)
Range	4–15	7–16	8–21	4–15	7–16	8–21	9–15	12–16	8–21
Classroom climate (whole questionnaire)									
M (SD)	101.86 (13.29	102.5 (13.87)	102.68 (14.07)	101.30 (14.05)	101.84 (13.68)	99.23 (14.30)	102.28 (12.75)	103 (14.07)	105.10 (13.47)
Range	49–125	42–128	45–128	53–125	42–121	45–124	49–123	47–128	67–128
Classroom climate (peer relationships)									
M (SD)	42.72 (6.17)	42.78 (6.08)	42.94 (6.31)	42.47 (5.84)	43.12 (6.78)	42.13 (6.78)	42.91 (6.43)	42.52 (6.24)	43.50 (5.94)
Range	20–52	18–52	16–52	20–50	18–52	16–52	21–52	23–52	24–52
Social skills									
M (SD)	3.89 (.62)	3.94 (.68)	3.93 (.71)	3.86 (.63)	3.85 (.70)	3.77 (.66)	3.91 (.62)	4.01 (.65)	4.04 (.72)
Range	1.91–5	2.07–5	1.55–5	1.91–4.84	2.07–5.	2.57–5.	1.91–5	2.55–5	1.55–5
Level of education (mothers): 1 –unfinished primary education to 10 –PhD									
M (SD)	7.64 (1.88)			7.70 (2.05)			7.60 (1.76)		
Range	2–10			2–10			2–9		
Level of education (fathers): 1 –unfinished primary education to 10 –PhD									
M (SD)	7.12 (2.06)			7.20 (2.00)			7.06 (2.11)		
Range	1–10			2–10			1–9		

We used repeated measures ANOVA to determine whether participants’ social, emotional, and motivational integration with school, academic (math and language) abilities, social skills, and classroom climate differed according to educational setting (the group variable) and time. There were no temporal changes in classroom climate in the case of peer interactions (time: *F*(2, 142) = 0.11; *p* = .89; time x group: *F*(2, 142) = 1.72; *p* = .18; results for all statement in the Climate of Inclusive Classroom questionnaire were: time: *F*(2, 142) = 0.37, *p* = 0.69; time x group: *F*(2, 142) = 2.15; *p* = 0.12); social integration (time: *F*(2, 142) = 0.02; *p* = .98; time x group: *F*(2, 142) = 0.63; *p* = .53); motivational integration (time: *F*(2, 142) = 0.08; *p* = .92; time x group: *F*(2, 142) = 1.75; *p* = .18); or social skills (time: *F*(2, 129) = 0.24; *p* = .78; time x group: *F*(2, 129) = 0.78; *p* = .46).

In the case of the remaining variables, there were changes over time, but no group differences were observed: emotional integration (time: *F*(2, 142) = 3.71; *p* < .05, η^*2*^ = 0.05; time x group: *F*(2, 142) = 2.41; *p* = .09); math (time: *F*(2, 143) = 234.46; *p* < .0001, η^*2*^ = 0.77; time x group: *F*(2, 143) = 0.21; *p* = .81); language (time: *F*(2, 128) = 57.13; *p* < .0001, η^*2*^ = 0.47; time x group: *F*(2, 128) = 0.18; *p* = .84). Moreover, we compared the level of education of mothers (*F*(1, 147) = 0.09; *p* = .75) and fathers (*F*(1, 137) = 0.15; *p* = .69) in both groups; the results indicated that groups did not differ also in this dimension. Overall, the results showed that there were no differences between the educational settings: almost all the dependent variables were stable over time or showed a similar pattern of change in the groups. In the case of one variable—emotional integration—scores in the general education classroom were lower at the second assessment than at the first assessment, but they did not differ between the third assessment and the first assessment. Both groups showed improvements in academic abilities over the course of the study. These results indicate that learning with children with disabilities did not have a negative impact on the classroom climate or the integration with school and peers, social skills, and academic achievement of children without disabilities.

### Main analysis

#### ToM Scale and Chocolate task

Table 2 presents the mean results for the ToM Scale. In the case of the ToM Scale, a linear model fit the data slightly better (PPP = 0.029; 95% confidence interval for the difference between the observed and replicated *chi*^*2*^ values was between -0.63 and 31.47; DIC = 1458.68) in comparison to the piecewise model (PPP = 0.022; 95% CI = 0.49 to 33.68; DIC = 1461.65). Thus, in next analyses, we used a model that assumed linear changes in ToM. In the description below we used following symbols: S_i_ = slope inclusive classroom; S_g_ = slope general classroom; I_i_ = intercept inclusive classroom; I_g_ = intercept general classroom; Δ = difference.

In inclusive classrooms, the intercept of the ToM result at the first assessment time (T1) was 4.22 (*SD* = 0.16; *p* < 0.05). The average change in ToM between T1 and T3 was positive and significantly different from 0 (S_i_ = 0.92; 95% CI = 0.74 to 1.11; *SD* = 0.10; *p* < 0.05). Therefore, in inclusive classrooms, there was a gradual increase in ToM abilities.

In general education classrooms, the intercept of ToM at the first assessment time was 4.46 (*SD* = 0.14; *p* < 0.05). There was no difference between groups in the case of the first assessment time (Δ_Ii-Ig_ = -0.24; 95% CI = -0.66 to 0.18; *SD* = 0.21; *p* > 0.05). Similarly, as in inclusive classrooms, in general education classrooms, there was a significant increase in ToM ability (S_g_ = 0.67; 95% CI = 0.51 to 0.82; *SD* = 0.08; *p* < 0.05). A between-group comparison showed that higher growth rates were characteristic of students from inclusive classrooms in comparison to those from general education classrooms (Δ_Si-Sg_ = 0.25; 95% CI = 0.01 to 0.50; *SD* = 0.12; *p* < 0.05. The analysis was also conducted with the following covariates: gender, level of parents’ education (a mean of mother’s and father’s level of education), social integration of children in classrooms, their social skills (ToPSS), and language achievement (all at T1, T2, T3). Results of the two analyses were similar.

**Fig 1:**
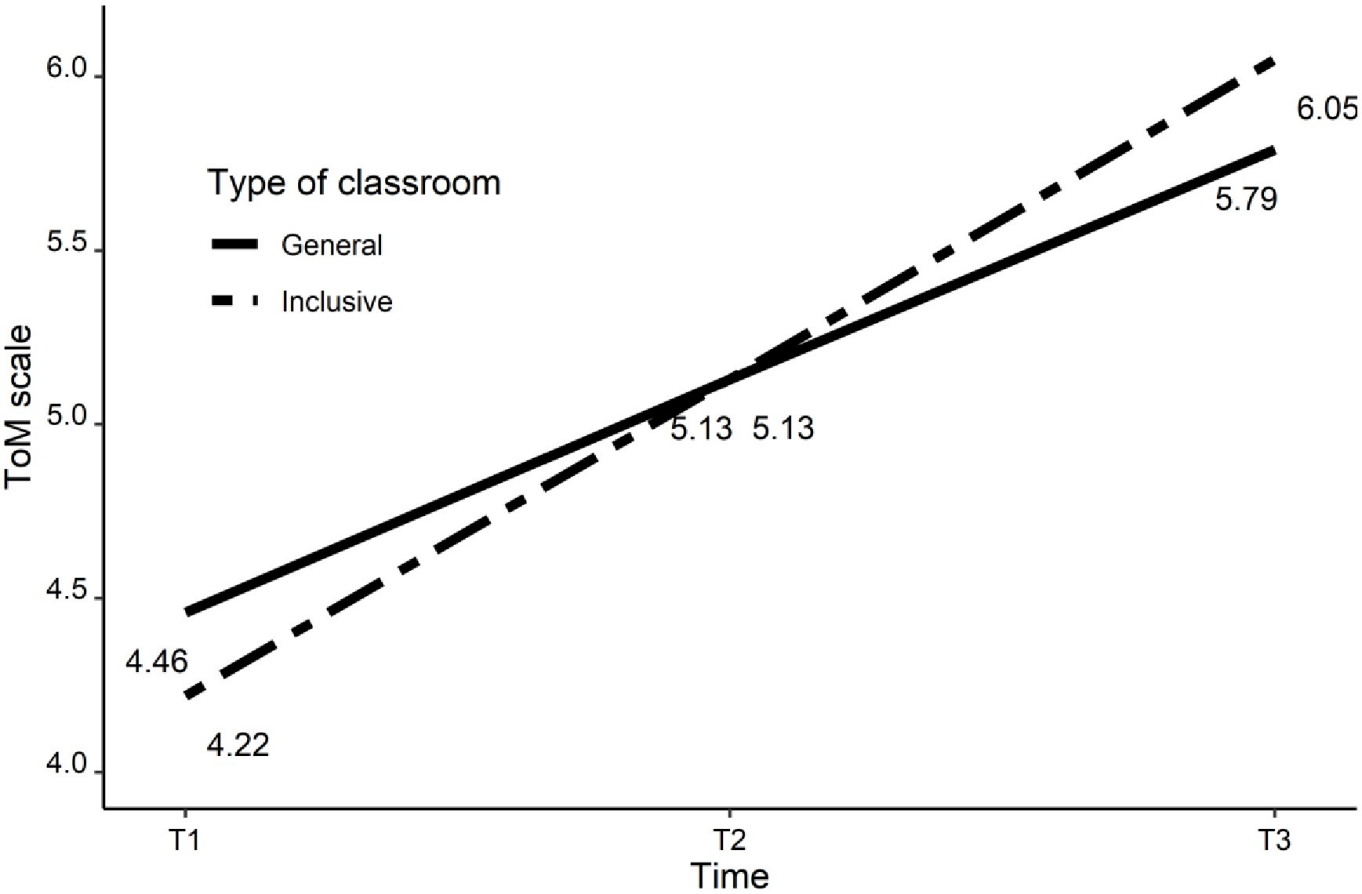
Changes in time in ToM Scale and Chocolate task scores.

Fig 1 illustrates the changes in the case of the ToM Scale scores (with the Chocolate task) for both groups of children.

#### Faux pas Recognition Test

Table 3 presents the mean results for the FPRT. The linear model fit for FPRT was as follows: PPP < 0.01; 95% CI = 20.33 to 52.14; DIC = 1863.16. The piecewise model was much better fitted to the data than the linear model (PPP = 0.340; 95% CI = -12.80 to 17.51; DIC = 1828.93). As a consequence, the next analyses were carried out with a model assuming nonlinear changes in the FPRT (the piecewise model).

**Table 3:** Mean results in the Faux Pas Recognition Test.

	Whole sample	Inclusive classroom	General education classroom
I assessment			
*M* (*SD*)	4.72 (2.09)	4.15 (1.78)	5.14 (2.20)
Range	0–10	0–7	0–10
Variance	4.35	3.18	4.86
II assessment			
*M* (*SD*)	6.33 (1.92)	6.22 (2.14)	6.42 (1.74)
Range	0–10	0–10	3–10
Variance	3.68	4.59	3.02
III assessment			
*M* (*SD*)	5.86 (1.63)	5.93 (1.60)	5.82 (1.67)
Range	2–10	2–9	2–10
Variance	2.69	2.56	2.80

The intercept had a value of 4.12 in inclusive classrooms (*SD* = 0.25, *p* < 0.05) and a value of 5.15 in general classrooms (*SD* = 0.21, *p* < 0.05). Students in inclusive classrooms obtained lower scores than students in general classrooms (Δi = -1.04; *SD* = 0.32; *p* < 0.05).

In the case of children from inclusive classrooms, there was a significant increase in the FPRT scores between T1 and T2, reflecting a significant mean difference between the two assessment times (S_1i_ = 2.081; 95% CI = 1.59 to 2.61; *SD* = 0.26; *p* < 0.05). Interestingly, for children in inclusive classrooms, there was no significant change in FPRT scores between the second and third assessment times (S_2i_ = -0.27; 95% CI = -0.71 to 0.15; *SD* = 0.22; *p* > 0.05). In general, however, during the entire study period (between T1 and T3) FPRT scores significantly increased for children in inclusive classrooms. The sum of slope coefficient piece 1 (S_1i_) and slope coefficient piece 2 (S_2i_) was positive and significant (SUM_S1i+S2i_ = 11.80; 95% CI = 1.29 to 2.42; *SD* = 0.29; *p* < 0.05).

**Fig 2:**
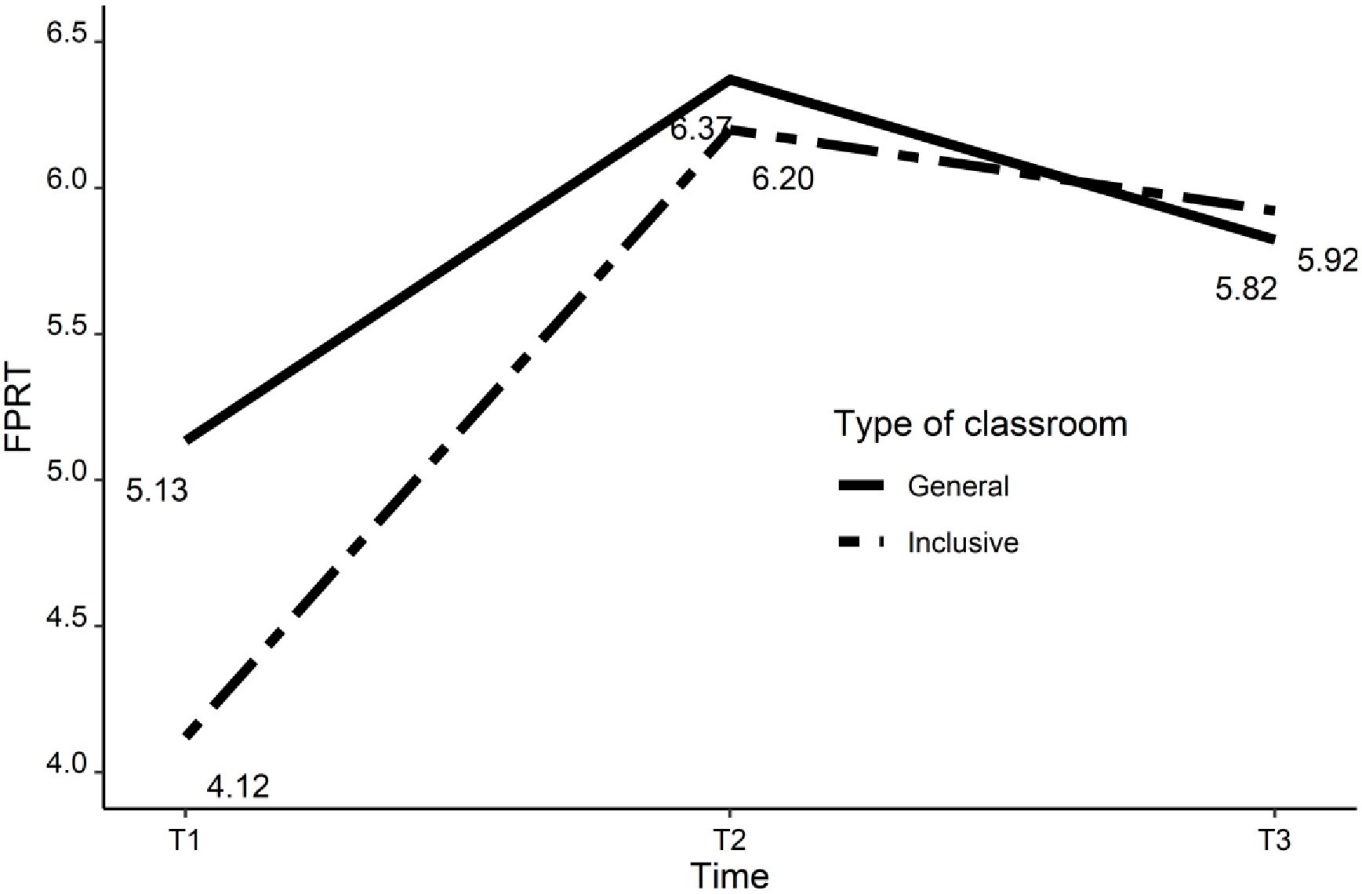
Changes over time in Faux Pas Recognition Test scores.

In the case of children in general education classrooms, changes in the FPRT scores looked slightly different. There were significant positive changes in the FPRT scores between T1 and T2 (S_1g_ = 1.22; 95% CI = 0.70 to 1.94; *SD* = 0.27; *p* < 0.05), but between T2 and T3, there was a significant decrease in scores (S_2g_ = -0.55; 95% CI = -1.02 to -0.11; *SD* = 0.24; *p* < 0.05). In general education classrooms, FPRT scores first increased but thereafter decreased. However, the sum of slope coefficient piece 1 (S_1g_) and slope coefficient piece 2 (S_2g_) was statistically significant (SUM_S1g+S2g_ = 0.67; 95% CI = 0.24 to 1.11; *SD* = 0.23; *p* < 0.05). This means that during the whole study (between time T1 and T3), FPRT scores increased; nonetheless, the changes were nonlinear (a visible increase at first and then a slight—but significant—decrease). Fig 2 illustrates the changes in the FPRT scores in both groups.

Using multigroup modeling makes it possible to compare changes in the growth rates of the FPRT scores for children in inclusive and general education classrooms. The change in the FPRT scores between T1 and T2 was larger in the case of children in inclusive classrooms than for children in general education classrooms (Δ_S1i-S1g_ = 0.86; 95% CI = 0.23 to 1.53; *SD* = 0.34; *p* < 0.05). However, there were no significant differences between the groups in terms of their FPRT scores between T2 and T3 (Δ_S2i-S2g_ = 0.27; 95% CI = -0.36 to 0.82; *SD* = 0.31; *p* > 0.05). Importantly, there was a significant difference between the groups with regard to the sum of changes in the FPRT scores between T1 and T3 (Δ_(S1i+S2i)-(S1g+S2g)_ = 1.14; 95% CI = 0.47 to 1.87; *SD* = 0.36; *p* < 0.05). This result indicates that throughout the entire studied period of time (between T1 and T3), FPRT scores increased more for children in inclusive classrooms than for children in general education classrooms. However, we must add that for the last assessment time (T3), children in the two groups were not significantly different from each other in terms of their FPRT scores (Δ_(Ii+S1i+S2i)-(Ig+S1g+S2g)_ = 0.10; 95% CI = -0.43 to 0.61; *SD* = 0.27; *p* > 0.05). Thus, faster changes in FPRT scores for children in inclusive classrooms led to the alignment of FPRT scores during the last assessment time in both groups. In the case of the analysis with FPRT scores, we also conducted a second analysis with covariates: gender, parents’ education, social integration of children in classrooms, their social skills (ToPSS), and language achievement (all at T1, T2, T3). Results of the two analyses were similar.

Generally, children who learned in inclusive classrooms had lower FPRT scores at the first assessment time than children in general education classrooms and exhibited a more visible increase in FPRT scores over the entire study period (between T1 and T3), primarily because of faster changes in FPRT scores between T1 and T2.

## Discussion

We carried out longitudinal observations to determine whether learning with children with disabilities is associated with enhanced ToM development in children without disabilities. We used three different measures for ToM: the ToM Scale with the Chocolate task and the FPRT. We hypothesized that being educated in an inclusive environment would be associated with faster ToM development. According to Carpendale and Lewis [27], ToM develops through triadic interactions relating to social values and social cognition, through reading about unknown ideas and through meeting people who are visibly different from us. It seemed probable that the greater understanding of diversity in inclusive classes, along with greater openness, tolerance and acceptance toward other children in the classroom and the use of prosocial behaviors, promotes ToM development. The results confirmed our hypotheses, and we made other interesting observations.

Our results confirmed the conclusion of other authors that children’s ToM continues to develop beyond the preschool years (e.g., [33, 57]). We observed positive changes in ToM in both groups in our study, and changes in ToM occurred more rapidly in the inclusive educational setting than in general education classrooms. In the case of the ToM Scale and Chocolate task scores, both groups developed ToM abilities over time; however, the changes observed for children in inclusive education classrooms were more visible in comparison to the changes observed for children in general education classrooms. In the case of the FPRT scores, we observed some interesting patterns in ToM development. At the beginning of the study, children in general education classrooms had significantly higher results for ToM than children in inclusive education classrooms. Nonetheless, during T1 and T2, children in both groups made significant progress. However, while children in the inclusive education setting did not change their scores between T2 and T3, children in general education classrooms experienced a significant decrease in scores during that period. Generally, during the whole study, the FPRT results for both groups increased, and there were no significant differences between the groups at T3. However, the positive changes observed for children in inclusive classrooms during the whole study were significantly larger than those observed for children in general education classrooms. To conclude, our results show that over time children in both groups presented a better understanding of first-order false belief (understanding what the other person can think, believe, want etc.), emotions of others, and meaning of sarcasm. They were also more often able to understand second-order false belief (understanding what the other person can think, believe, and know about a third person). Additionally, our results show that learning in an inclusive classroom can be beneficial for developing these abilities. However, in general, understanding of ambiguous situations and faux pas does not change notably over time regardless of the educational setting but rather is a somewhat stable ability.

It is very difficult to speculate about the causes of the nonlinear changes in FPRT scores for children in general education classrooms. Moreover, even though Baron-Cohen’s original research [37] showed that children’s results on the FPRT improved with age, the study by Hayward and Homer [4] did not find any age effect on this and other advanced ToM tests. It is important to mention that some research has shown nonlinear development in children (e.g., see [58]), including ToM [59], although this is not a common result [57]. Analysis of other classroom characteristics, such as classroom climate (contacts with peers), integration with peers and school, motivation to learn, academic achievement and children’s social skills, did not reveal any differences between the settings. The lack of group differences in the classroom variables that we analyzed suggests that they are not responsible for the positive role of inclusive education in ToM development. Despite the lack of differences in the results for variables other than ToM, and because the overall observed rate of changes was higher for children in inclusive classrooms than for children in general education classrooms, we further focus on possible causes of such results. First, we can refer to the “peer effect” [60, 61] and diversity in the classroom. We believe that children without disabilities who are educated in inclusive settings gain a better understanding of peers with disabilities and learn how to help them, which aids in developing prosocial behaviors. A relationship between ToM development and prosocial behaviors was reported in meta-analysis [28]. Other analyses of the “contact hypothesis” have shown that real-life contact with people who are different from us reduces prejudices toward others (e.g., [62]). According to the contact hypothesis, this is because contact with other people increases our knowledge of them, decreasing our fear of the unknown. Other studies have shown that a good atmosphere in the classroom can improve cohesion, trust and respect [63, 64]. In our study, the classroom atmosphere was similarly good in both educational settings, which can be interpreted as a point in favor of inclusive education: it appears that the permanent presence of children with disabilities does not influence the sense of integration of children without disabilities or their motivation, academic or social skills. Moreover, more visible improvement of ToM in inclusive settings is important and powerful evidence in favor of inclusive education.

The results of our study have important applications and consequences. The study shows that inclusive education can be associated with enhanced development of ToM among children without disabilities. Thus, this result can be a strong argument for implementing this educational concept. Our results particularly highlight the fact that a more heterogeneous environment and constant contact with children with disabilities can foster social cognition and understanding of others. Therefore, in situations in which inclusive education is impossible, it is worth providing children chances to meet peers with disabilities, allowing them to spend time together and in this way learn more about others’ minds. It is equally important to note that ToM has a predictive role in children’s future functioning, and it is therefore important to identify factors that positively influence its development. Longitudinal research has shown that children with better developed ToM are more often accepted by peers [65, 66], more often display prosocial behaviors (e.g., [67]) and are less aggressive than peers with less developed ToM [68]. Moreover, ToM is predictive for academic achievement—children with better ToM have higher academic achievement [69–71].

Although our study has several strengths, including its longitudinal design and fairly large sample, it also has some weaknesses.

We were unable to determine which classroom characteristics were responsible for the interesting results we observed. It is possible that the positive effect that inclusive education had in our study is not simply a diversity effect but also due to the ways in which inclusive classes are taught. Teachers in inclusive classes might engage in perspective taking while communicating with children, give special attention to empathy toward others, and support cognitive decentering more often in comparison to those teaching in general education classrooms. However, these differences are related to the more diverse environment of the inclusive classroom and the more diverse needs of children learning in these classrooms. Therefore, the observed differences between educational environments are not only a simple function of interactions with children with disabilities, but also reflect other elements associated with the presence of children with special needs in classrooms. Other studies conducted in school environments have shown that paying attention to the emotions of characters in stories, practicing mental verbs and encouraging children to discuss different social situations all have a beneficial effect on ToM development [11–14]. Observing lessons would provide valuable information about teaching methods and the learning process in different educational environments. It is possible that the teachers of inclusive classes use different techniques from their colleagues teaching general education classes to capture the attention of their diverse students and transmit knowledge effectively to children with and without disabilities. Unfortunately, it was impossible for us to collect observational data about differences in teaching techniques during this study because the data were collected from schools throughout the country. In the future, however, it would be worth investigating whether there is an association between teaching methods and ToM development in smaller groups of children studying in their natural school environment. The data collection approach and the design of the study had some consequences: children were not randomly assigned to groups, which resulted in children in general education classrooms having slightly higher results on the FPRT at the first assessment time than children in inclusive classrooms.

It is probable that the intervals between the assessment times were too short; however, it was impossible to make them longer because of the project requirements. We could also speculate whether weaker changes in ToM over time (but not the overall result) in general education classrooms are not connected to regression to the mean. Even if we cannot eliminate this cause from our results with certainty, it seems unlikely because the children’s results were not even close to the ceiling. However, we cannot exclude the possibility of practice effect among our participants. Although this effect could have been present, the possibility is not high. Some tasks were repeated, but many were not. Additionally, intervals of 10 months between waves seem to provide sufficient time for participants to forget the tasks, and even though the children remembered some tasks, they did not know the correct answers. Additionally, in both groups, the differences between the first and last assessment times were significantly positive and similar to one another. The pattern of changes was similar; only its strength was different.

## Conclusion

Our study adds to the existing experimental research on ToM in the school environment [11–14]. It shows that educational setting, i.e., whether or not classes include children with disabilities can be associated with enhanced ToM development in children without disabilities. We observed that in children without disabilities, ToM development is more visible in an inclusive educational environment than in a general educational environment from which children with disabilities are absent. This result not only improves knowledge of ToM development in the school environment but also provides further evidence that inclusive education can be beneficial for *all* children, not only in the case of academic achievement [16–18], but also for other cognitive abilities, such as ToM. Our study broadens knowledge about the social factors that influence ToM and shows that for children in middle childhood, school is an important milieu for ToM development.

## Supporting information

S1 Data (SAV)
